# Combined metabolic analyses for the biosynthesis pathway of l-threonine in *Escherichia coli*

**DOI:** 10.3389/fbioe.2022.1010931

**Published:** 2022-09-09

**Authors:** Qiang Yang, Dongbo Cai, Wenshou Chen, Huiying Chen, Wei Luo

**Affiliations:** ^1^ The Key Laboratory of Industrial Biotechnology, Ministry of Education, School of Biotechnology, Jiangnan University, Wuxi, China; ^2^ State Key Laboratory of Biocatalysis and Enzyme Engineering, Environmental Microbial Technology Center of Hubei Province, College of Life Sciences, Hubei University, Wuhan, China; ^3^ College of Chemistry and Bioengineering, Guilin University of Technology, Guilin, China

**Keywords:** Escherichia coli, l-threonine, combined metabolic analyses, metabolic flow, enzyme control analysis, metabolomics

## Abstract

Currently, industrial production of l-threonine (Thr) is based on direct fermentation with microorganisms such as *Escherichia coli*, which has the characteristics of low cost and high productivity. In order to elucidate the key metabolic features of the synthesis pathway of Thr in *E. coli* to provide clues for metabolic regulation or engineering of the strain, this study was carried out on an l-threonine over-producing strain, in terms of analyses of metabolic flux, enzyme control and metabonomics. Since environmental disturbance and genetic modification are considered to be two important methods of metabolic analysis, addition of phosphate in the media and comparison of strains with different genotypes were selected as the two candidates due to their significant influence in the biosynthesis of Thr. Some important targets including key nodes, enzymes and biomarkers were identified, which may provide target sites for rational design through engineering the Thrproducing strain. Finally, metabolic regulation aimed at one biomarker identified in this study was set as an example, which confirms that combined metabolic analyses may guide to improve the production of threonine in *E. coli*.

## Introduction

l-threonine (Thr) is one of the eight essential amino acids and mainly used in food fortification, pharmaceuticals, chemical reagents and feed additives (Komatsubara et al., 1978; Follettie et al., 1988; Leuchtenberger et al., 2005), with the amount used in feed additives in particular growing rapidly (Zheng et al., 2020). It is often added to the feed of immature piglets and poultry and is the second limiting amino acid in pig feed and the third limiting amino acid in poultry feed. The current methods of producing Thr mainly include hydrolysis of animal proteins and microbial fermentation. Compared with the former, the microbial fermentation method has the advantages of low cost and low pollution (Li et al., 2021;). Among the Thr-producing strains, *E. coli* is the main host, which is ascribed to its simple genetic background and convenience to modify through metabolic engineering (Debabov et al., 1981; Song et al., 2000). In addition, it is of short growth cycle, high cell intensity and low requirement for equipments. Efficient synthesis of Thr does not only depend on a large increase in the efficiency of a particular rate-limiting reaction, but requires a balance of multiple metabolic pathways in the biosynthetic network, such as intensification of the target metabolite flow, weakening the competitive branch metabolite flow, improvement of the extracellular transport efficiency, etc. (Dong et al., 2011; Lee et al., 2009).

Metabolic flux analysis (MFA) is a metabolic network analysis method via the intracellular reaction stoichiometry model, which is based on the pseudo-steady state assumption that the metabolic flux distribution of all metabolites is estimated in a dynamic equilibrium between the rates of production and consumption in the metabolic pathway (Lee et al., 2003; Park and Lee, 2010). This method has been successfully applied to the metabolic analyses of some amino acids and has resulted in significant savings in experimental costs. Since metabolite synthesis in the metabolic pathway is controlled by various enzyme-catalyzed reactions, it is necessary to investigate the control effect of these enzymes on carbon flux. Metabolic control theory believes that the change of metabolic pathway reaction at each step will cause the change of system parameters, so there is no stable rate-limiting step for biochemical reaction in metabolic pathway (Toya et al., 2011; Wang et al., 2014; Zhu et al., 2021). Flux control coefficient (FCC) and elasticity coefficient constitute the genetic theory of metabolic control analysis, which describe the global parameters in the whole metabolic network, and are also the bridge between enzymatic activities and metabolic fluxes. Another method for cell metabolic analysis relies on metabonomic technology, which detects the change of metabolites through GC/MS or LC/MS and accurately reflects the metabolic change under different conditions (Yang et al., 2018; Park et al., 2021). Metabonomics research now penetrated into many fields, such as medicine (Schmid et al., 2004; Antoniewicz, 2021), food (Picone et al., 2011; Antoniewicz, 2021) and microbiology (Luo et al., 2020).

In this study, phosphate was observed as a significant factor affecting the biosynthesis of Thr (Liu et al., 2020), thus was selected as an environment disturber to elucidate the biosynthesis pathway by metabolic flux analysis. The effect of phosphate on the metabolic flux distribution of Thr biosynthesis was investigated under fed-batch mode. On the other hand, the enzyme activities involved in Thr synthesis were measured and the metabolic control coefficients on fluxes were calculated by multivariate statistical analysis, which may provide a theoretical basis for the modification and metabolic regulation of key enzymes. Furthermore, the influence of genetic modification on the synthesis pathway of Thr through metabonomic analysis was investigated. The differential metabolites and metabolic pathways were observed in two strains with different genotypes and some biomarkers were obtained through multivariate statistical methods. Then metabolic regulation on a target obtained from the above metabolic pathway analyses was set as an example to improve the production of Thr.

## Materials and methods

### Strains, reagents and instruments

Thr over-producing srain *E. coli* TWF001 was used for the metabolic analyses for the biosynthesis pathway of Thr and W3110 was used as a control (Zhao et al., 2018). Methanol and acetonitrile with chromatographic grade were used as mobile phase in the determination by high performance liquid chromatography (HPLC). Pre-column derivatizer o-phthalaldehyde, sodium hydroxide, ammonia and 10% trichloroacetic acid were purchased from local market. Biosensor SBA-40C (Shandong Academy of Sciences, China) and Agilent HPLC were used for determination of glucose and other metabolites, respectively.

### Culture media

Basic seed medium contained 10 g L^−1^ peptone, 5 g L^−1^ yeast extract and 10 g L^−1^ sodium chloride, which was adjusted to pH 7.0 and sterilised at 115°C for 30 min. Initial fermentation media (Zhao et al., 2020) contained 20 g L^−1^ glucose, 6.4 g L^−1^ beet molasses, 3 g L^−1^ maize pulp, 0.7 g L^−1^ betaine HCl, 0.4 g L^−1^ MgSO_4_, 0.9 g L^−1^KCl, 0.011 g L^−1^ MnSO_4_·H_2_O, 0.011 g L^−1^ FeSO_4_·7H_2_O and 0.9 g L^−1^ H_3_PO_4_ at pH 7.0.

### Seed culture and l-threonine fermentation

The conserved strains from the tube were picked out, streaked onto the prepared activation medium slant, and incubated at 37°C for 8–10 h a 250 ml flask supplemented with 50 ml of seed medium, which was incubated at 37 °C for 14 h in a shaker with 200 r·min^−1^. For batch fermentation, 250 ml shake flasks supplemented with 50 ml of fermentation medium were inoculated with seed culture at 1% pitching rate and incubated at 37°C for 48 h in a shaker with 200 r min^−1^.

Fed-batch fermentation of Thr was conducted at 37°C in a 5 L fermentor by continuous feeding 500 g L^−1^ glucose to maintain its concentration between 5 and 20 g L^−1^. Dissolved oxygen was adjusted by ventilation rate and agitation speed at around 30% of the solubility saturation and pH was maintained at between 6.8 and 7.2 with 25% ammonia (Wang et al., 2014).

### Metabolic flow analysis

According to the literatures (Park and Lee, 2010; Su et al., 2018), Embden-Meyerhof-Parnas (EMP), tricarboxylic acid (TCA), hexose monophosphate pathway (HMP), anaplerotic route and phosphotransferase system (PTS) are present in *E. coli*. HMP pathway is important for amino acid synthesis since large amounts of reducing substances NADH/NADPH are produced to maintain cytoplasmic redox equilibrium. In addition, the glyoxalate cycle does not occur in *E. coli* when glucose is used as the substrate, indicating that the TCA cycle is still the main oxidative pathway in *E. coli*. Therefore, pathways of EMP, HMP and TCA were set as the main network pathways when constructing the Thr metabolic network.

**FIGURE 1:**
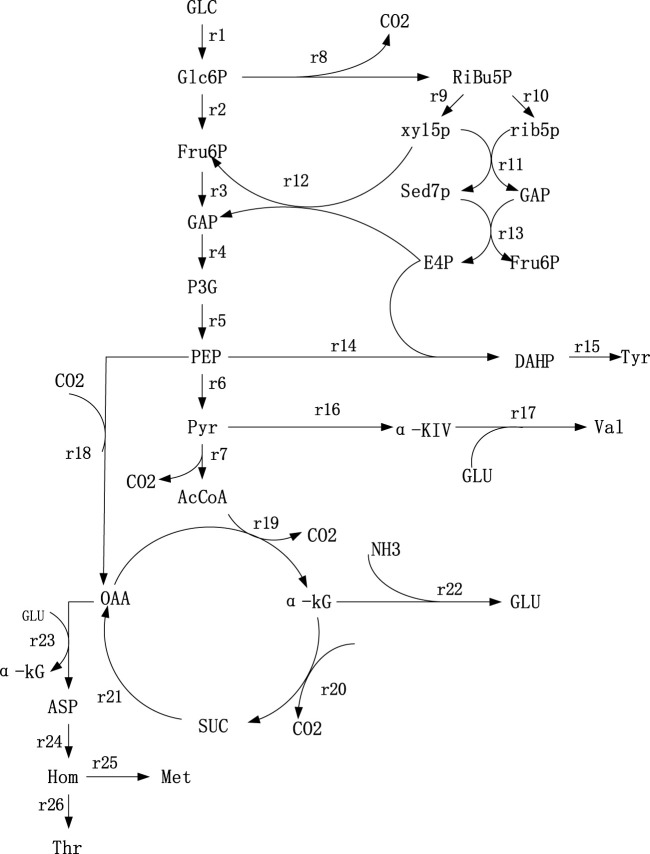
Metabolic network of Thr synthesis. 1) Glc: Glucose; 2) Glc6P: Glucose-6-phosphate; 3) PEP: Phosphoenolpyruvate; 4) Pyr: Pyruvate; 5) Fru6P: Fructose- 6-phosphate; 6) GAP: Glyceradehyde-3-phosphate; 7) P3G: 3-phosphoglycerate; 8) AcCoA: AcetylcoenzymeA; 9) Ribu5P: Ribulose-5-phosphate; (10) Xyl5P: Xylulose-5-phosphate; 11) Rib5P: Ribose-5-phosphate; 12) Sed7P: Sedoheptulose-7-phosphate; 13) E4P: Erythrose-4-phosphate; 14) OAA: Qxaloacetate; 15) α-KG: α-ketoglutarate; 16) NADPH: Nicotinamide adenine dinu-cleotide phosphate; 17) Glu: Glutamate; 18) αKiv: α-ketoisovalerate; 19) Asp: Aspartate; (20) Hom: Homoserine; 21) DAHP: 3-deoxy-d-arabi-noheptulosonate-7-phosphate.

According to Thr synthesis pathway in the KEGG database, some references (Schmid et al., 2004; OlafKrömera et al., 2006) and the metabolites detected in strains TWF001, the metabolic network was established based on the following principles, 1) Cells in the period of pseudo-steady state are of non-growth and the biomass changes can be ignored; 2) the total amount of NADPH consumed in the reaction pathway is equal to the total amount of NADPH produced by the TCA cycle and the HMP pathway, i.e. the balance of NADPH supply and demand; 3) the glyoxalate cycle does not exist during cell metabolism; 4) reactions that proceed in a fixed ratio and intermediate reactions without branching points are simplified to a reaction equation; 5) during the stagnation phase of cell growth, the total cellular maintenance energy is not equal to the ATP consumption due to the presence of a large number of invalid cycles, so the balance of total ATP is not considered; the network of Thr biosynthesis metabolism is shown in Figure 1.

According to the assumption that the intermediate metabolites is in the pseudo-steady state, the change rate of intracellular metabolites is 0. The accumulation rate of metabolites is calculated according to the following equation,

$$r_{i}\left(t\right)=\sum_{i=1}^{M}\partial_{ij}r_{j}\left(t\right)-\sum_{i=1}^{i=N}\partial_{ik}r_{k}\left(t\right)$$

In this rate equation, r_i_(t) is the rate of accumulation of intermediate metabolite i (mmol·(Lh)^−1^); r_j_(t) is the reaction rate of the reaction step j for the synthesis of i (mmol·(Lh)^−1^]; r_k_(t) is the reaction rate of the reaction step k for the synthesis of i (mmol·(Lh)^−1^); ∂_*ij*_ is the stoichiometry coefficient of the reaction step j; ∂_*ik*_ is the stoichiometry coefficient of the reaction step k. The rate equations at the metabolite nodes in the metabolic network were shown in Supplementary Table S2. The set of rate equations constructed consists of 21 equations with 26 unknowns and a degree of freedom of 5. The contents of glucose, Thr, l-tyrosine (Tyr), l-valine (Val) and l-methionine (Met) were measured, which were used as known parameters and substituted into the above set of metabolic rate equations to obtain the metabolic flow distribution using lingo software.

### Determination of enzyme activities

When the cell growth entered stationary phase (under pseudo-steady state), samples were collected and centrifuged at 10,000 rpm for 5 min in a refrigerated centrifuge at 4°C. 0.5 g wet weight of the cells was washed by 10 ml of sterile 0.01 M PBS phosphate buffer for twice. Then 10 ml enzyme extraction solution were added for sonication and fragmentation treatment was under the following conditions, sonication power (PW) of 80%, working time of 2 s, intervals of 1 s, repeated 30 times. The samples were then centrifuged at 4°C for 5 min at 8,000 rpm to remove the precipitates and the enzyme solution was kept at a low temperature before determination of enzyme activities. The activities of pyruvate kinase (PK), malate dehydrogenase (MDH), fructose 1,6-diphosphate aldolase (FBA), glucose-6-phosphate dehydrogenase (G6PD), phosphoenolpyruvate carboxylase (PEPC) and hexokinase (HK) were determined by kits purchased from Solarbio Biotechnology Ltd. Protein concentrations were measured using the Coomassie Brilliant Blue G250 staining method at 595 nm and protein content was detected according to the increase in peak absorption.

### Principal component analysis

For correlation analysis of the activities of different enzymes in the Thr synthesis pathway, the complex, multidimensional nature of the data requires the use of a multivariate data analysis method, i.e., principal component analysis (Antoniewicz, 2021), when the correlation between variables is significant. The six enzyme activities in the Thr synthesis pathway were measured under fed-batch mode and submitted to principal component analysis, which were set as initial indicators and noted as in order, X1 to X6. The screening conditions for principal component analysis were set as follows, the eigenvalues *λ* > 1, the principal components cumulatively reflecting more than 85% of the original information. Using SPSS25.0 software, the equation F_j_ = a_1j_X_1_+a_2j_X_2_......+a_10j_X_10_ (j = 1, 2, 3......10) was obtained, where the coefficients of the expression of the principal component reflect the combined influence of each enzyme on the principal (Vo et al., 2018). This analysis excludes the effect of enzyme synergy on Thr flux, as the principal component eliminates the correlation of the initial indicators.

### Metabolism quenching and metabolites pre-treatment

Samples were collected and centrifugated at 4°C, 10,000 rpm for 5 min to remove the precipitates, and the supernatant was mixed with cold methanol with a volume ratio of 1:3. The mixes were shaked and precipitates occurred during this process were removed by centrifugation at 4°C, 10,000 rpm for 5 min. All samples were kept in an ice box throughout the operation and this process was repeated twice.

The extracted metabolites were quantified using an internal standard method (Luo et al., 2020) and the detailed operation steps are as follows. 500 µL of the extract were added with 30 µL of the internal standard reagent ribitol (1 mg mL^−1^), and the mixes were shaken well and blown dry in a nitrogen blower. Then derivatization of metabolites was conducted by reactions of oximation and silylation. For the former, the samples were mixed with 350 µL of pyridinium methoxide hydrochloride solution (20 mg mL^−1^) for 2 h at 30°C. Afterwards, 350 µL of BSTFA-TMCS (99:1, V/V) solution were added into the solution and the reaction was carried out at 70°C for 1 h. Then samples were blown dry with a nitrogen blower, and 1 ml of hexane/dichloroacetic acid was added to re-dissolve them. The samples were treated with a 0.22 µm filter membrane and detected on a gas chromatograph-mass spectrometer (GC-MS) machine (Jonsson et al., 2004; Koek et al., 2010).

### Gas chromatograph-mass spectrometer condition

Metabolomic detection was conducted on Thermo GC-MS (TSQ8000; Thermo Scientific, United States) with a column (TG-5, 30*0.25 mm*0.25 µm), using a helium carrier gas at a flow rate of 1.2 ml min^−1^ and operating at splitting ratio of 5.0. Electron energy and emission current were set to 70 eV and 25 μA, respectively. GC oven temperature was raised to 65°C, maintained for 2 min and then increased to 280°C at a constant speed in 13 min. Interface, transmission line and ion-source were kept at 300°C, 280°C and 300°C, respectively. The mass spectral scan range was set at 35–650 mz.

### Data analyses

The metabolites were qualitatively analyzed by searching the spectrum in the GC-MS database or comparison with standards, and the relative contents of metabolites were determined according to the peak area of the internal standard ribitol in the solution. The data obtained were collated into csv format and imported into SIMCA-P software for principal component analysis and discriminant analysis. Cluster analysis made by Origin software was used to resolve intra- and inter-group relationship.

### Metabolite determination by high performance liquid chromatography and biosensor

Samples were centrifuged at 10,000 rpm for 2 min to remove the precipitates and then treated with 10% trichloroacetic acid at equal volume for 4–8 h to remove proteins from the samples.

The determination of Thr and other amino acids was carried out by HPLC with a C18 column (5 μm, 250 mm × 4.6 mm) (Luo et al., 2021). The mobile phase was divided into two parts, A and B. The mobile phase A contained 10 mmoL·L^−1^ disodium hydrogen phosphate and sodium tetraborate, and the mobile phase B consisted of methanol: acetonitrile: water = 45:45:10 (v/v/v). The gradient elution program was (volume ratio of mobile phase B) 5% for 0–0.35 min, 5%–57% for 0.35–13.40 min, 57%–100% for 13.40–13.50 min, 100% for 13.50–15.70 min, 100–5% for 15.70–15.80 min and 5% for 15.80–18 min at 40°C with a flow rate of 1 ml min^−1^ and a detection wavelength of 338 nm.

Glucose concentration was determined using a biosensor (Su et al., 2018).

## Results and discussion

### Effect of phosphate concentration on l-threonine synthesis in fed-batch culture mode

From the results of the PB experiments in shake flask, phosphate is the most significant factor affecting Thr synthesis (Supplementary Table S3). In order to more truly show the effect of phosphate on Thr synthesis, effect of addition of phosphate on Thr accumulation in 5 L fermentor was investigated.

**FIGURE 2:**
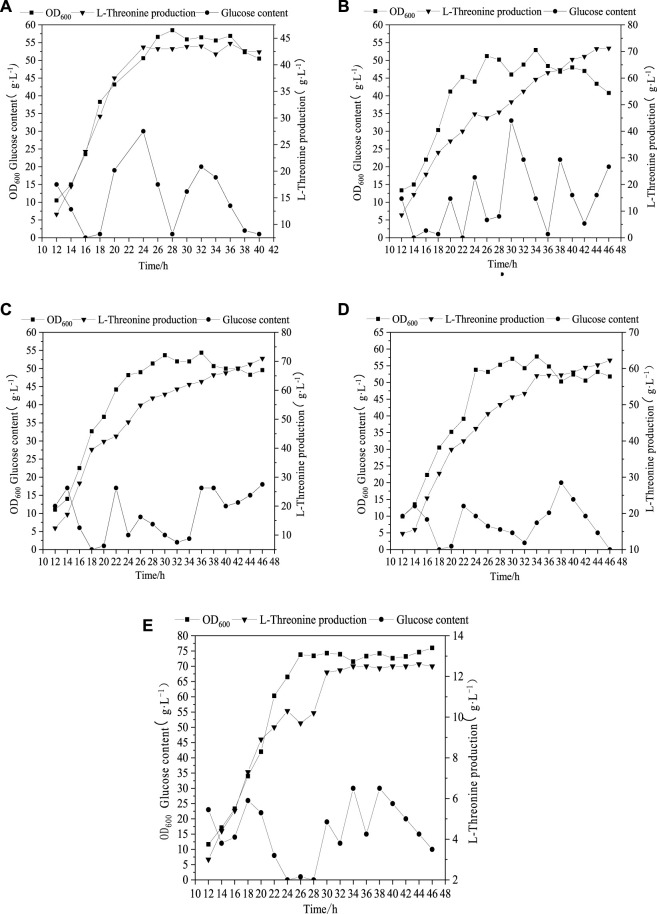
Profile of Thr production under different initial phosphate concentration. **(A)** (4.8 g L^−1^), **(B)** (9.8 g L^−1^), **(C)** (14.8 g L^−1^), **(D)** (19.8 g L^−1^), **(E)** (24.8 g L^−1^).

As can be seen from Figure 2A, when 4.8 g L^−1^ phosphate was added, the maximum biomass (OD_600_) and Thr production were 56 and 42.3 g L^−1^, respectively. With the increment of phosphate amounts up to 24.8 g L^−1^, the biomass (OD_600_) was improved to 76, while the change of Thr did not keep increasing (Figure 2E). The optimal concentration range of phosphate for the maximum Thr production is 9.8–14.8 g L^−1^ (Figures 2A–E), which indicates that the increased phosphate concentration may cause the migrating of C metabolic flow to the growth of biomass rather than the increase of target products. Phosphorus element is one of the core elements in microbial growth and metabolism (Anandan et al., 2014), which is involved in the composition of nucleic acids, cell membranes and high-energy phosphate compounds in life activities, and is also an important player in central metabolic pathways (Lai et al., 2012). The present study confirms that the addition of phosphate promotes the growth of cell and affects the accumulation of Thr. However, the details of phosphorus affecting metabolic flux are not very clear, which should be solved by determination of the metabolic flow distrubution.

### Calculation of the metabolic flow at different phosphate concentration

Cells were cultured under fed-batch mode with different initial phosphate concentration and samples were collected at the pseudo-steady state period. Contents of glucose, Thr, L-Val, L-Tyr and L-Met were measured and the rates of metabolite consumption and accumulation were calculated as shown in Supplementary Table S4. Lingo software was used to estimate the metabolic flow distribution in the metabolic network and linear programming was performed in Excel to obtain the ideal metabolic flow distribution for Thr biosynthesis (Table 1). It can be calculated that the conversion rates of glucose to Thr are 45.4% under 9.8 g L^−1^ phosphate and 27.9% under 24.8 g L^−1^ phosphate, which are much lower than that value (73.3%) under ideal condition. With the increase of phosphate concentration, the flow rate on the branch of Thr synthesis decreases, causing the increase of by-products.

**TABLE 1:** Metabolic flow distribution of Thr biosynthesis network.

Reaction no.	Metabolic flux distribution calculated [mmol·(L h)^−1^]		Theoretical metabolic flux distribution [mmol·(L h)^−1^]	Reaction no.	Metabolic flux distribution calculated [mmol·(L h)^−1^]		Theoretical metabolic flux distribution [mmol·(L h)^−1^]
	9.80 g L^−1^	24.80 g L^−1^			9.80 g L^−1^	24.80 g L^−1^	
r1	100.00	100.00	100.00	r14	1.08	7.083	0
r2	75.40	84.79	0	r15	1.08	7.083	0
r3	91.44	92.57	66.67	r16	0.16	9.72	0
r4	181.80	178.06	166.67	r17	0.16	9.72	0
r5	181.80	178.06	166.67	r18	46.05	44.17	73.33
r6	134.67	126.81	93.33	r19	134.52	117.08	93.33
r7	134.52	117.08	93.33	r20	134.36	107.36	93.33
r8	24.60	15.208	100	r21	134.36	107.36	93.33
r9	16.04	7.78	66.67	r22	46.05	44.17	73.33
r10	8.56	7.43	33.33	r23	45.89	34.44	73.33
r11	8.56	7.43	33.33	r24	45.89	34.44	73.33
r12	7.48	0.35	33.33	r25	0.54	6.53	0
r13	8.56	7.43	33.33	r26	45.35	27.92	73.33

### Metabolic flow analysis of key nodes in l-threonine synthesis pathway

**FIGURE 3:**
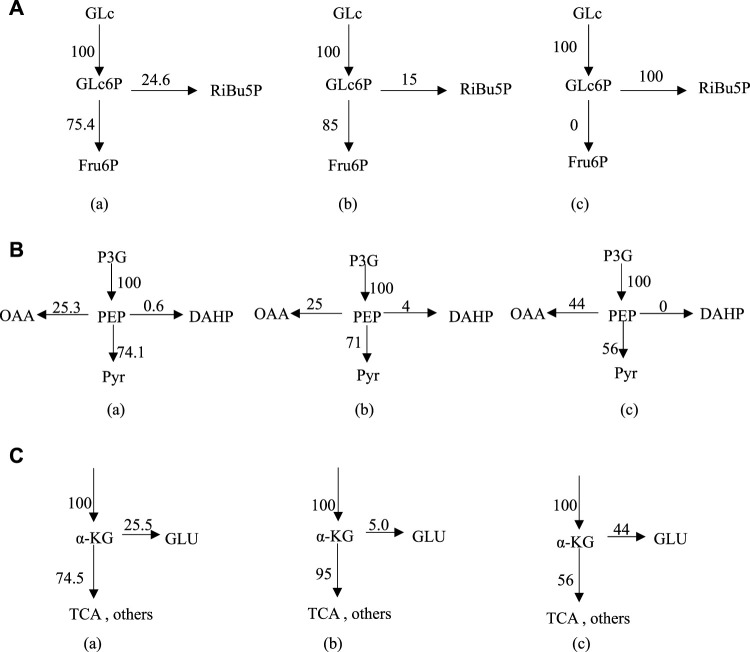
Metabolic flux distribution at Glc6P node **(A)**, PEP node **(B)** and α-KG node **(C)** under different conditions; **(A)** 9.8 g L^−1^ phosphate, **(B)** 24.8 g L^−1^ phosphate, **(C)** ideal condition. Enzyme control analysis for metabolic flux in Thr biosynthesis network.

At Glc6P nodes, when phosphate concentration changed from 9.8 g L^−1^–24.8 g L^−1^, the r8 flow [C/mmol·(L h)^−1^] to the HMP pathway decreased from 24.6 to 15, while the r2 flow [C/mmol·(L h)^−1^] increased from 75.4 to 85 (Figure 3A). Considering the ideal metabolic flow (r2 = 0), it is inferred that enhancement of the HMP pathway metabolic flow can lead to an increase in the target metabolic flow (r26), which causes the conversion rate of glucose to Thr increased from 28% to 45%. At PEP node (Figure 3B), 74.1% and 71% of the carbon flux (100% PEP) went to Pyr in the presence of phosphate at 9.8 g L^−1^ and 24.8 g L^−1^, respectively. However, metabolic flow distribution under the ideal condition is 44% of the carbon flux going into OAA and 56% going to the Pyr, indicating that reduction of the flux to the TCA may help improve the synthesis of Thr. An increase in the metabolic flow of aromatic amino acid synthesis through overexpression of phosphoenolpyruvate synthase has been reported (FARMER and LIAO, 1997), thereby indirectly increasing Thr production and reducing by-product production. From Figure 3C, it can be seen that 25.5% and 5% of α-KG are catalyzed by l-glutamate dehydrogenase and l-glutamate synthase to form l-glutamate in the presence of phosphate at 9.8 g L^−1^ and 24.8 g L^−1^, respectively. The remaining 74.5% and 95% of α-KG enters the TCA cycle under the above conditions, respectively. However, 56% metabolic flow enters the TCA cycle (r20) and 44% is used for the synthesis of l-glutamate (r22) under the ideal condition. Thus, l-glutamate is a key intermediate to provide sufficient substrate for the transamination required for the synthesis of the aspartate group of amino acids, the precursor of Thr.

Firstly, correlation analysis was performed for the six enzyme in Thr biosynthesis network and correlation coefficients were calculated for them (Table 2). The correlation coefficient between PK and PEPC is 0.915, which shows statistically significant.

**TABLE 2:** Correlation analyses between variable enzymes.

Enzyme		PEPC	HK	FBA	MDH	PK	G6PD
PEPC	Pearson correlation coefficient	1	−0.706	−0.066	−0.421	0.915^a^	0.776
	Sig. (2-tailed)		0.117	0.901	0.405	0.010	0.070
	Number	6	6	6	6	6	6
HK	Pearson correlation coefficient	−0.706	1	0.008	0.602	−0.673	−0.390
	Sig. (2-tailed)	0.117		0.989	0.206	0.143	0.445
	Number	6	6	6	6	6	6
FBA	Pearson correlation coefficient	−0.066	0.008	1	0.669	−0.133	−0.077
	Sig. (2-tailed)	0.901	0.989		0.146	0.801	0.885
	Number	6	6	6	6	6	6
MDH	Pearson correlation coefficient	−0.421	0.602	0.669	1	−0.370	0.019
	Sig. (2-tailed)	0.405	0.206	0.146		0.470	0.972
	Number	6	6	6	6	6	6
PK	Pearson correlation coefficient	0.915^a^	−0.673	−0.133	−0.370	1	0.804
	Sig. (2-tailed)	0.010	0.143	0.801	0.470		0.054
	Number	6	6	6	6	6	6
G6PD	Pearson correlation coefficient	0.776	−0.390	−0.077	0.019	0.804	1
	Sig. (2-tailed)	0.070	0.445	0.885	0.972	0.054	
	Number	6	6	6	6	6	6

a Denotes significance at 0.05 level (two-tailed).

To avoid the overlap of information between these enzymes, their activities of six enzymes selected in Thr metabolic network were subjected to principal component analysis (Table 3). The cumulative contribution of the first three principal components reached 95.9%, thus they are able to represent the information of all the initial indicators.

**TABLE 3:** Principal ingredient extraction.

Ingredients	Initial eigenvalue			Extraction of sum of squares of loads		
	Total	Percentage variance	Cumulative %	Total	Percentage variance	Cumulative %
1	3.408	56.799	56.799	3.408	56.799	56.799
2	1.549	25.824	82.623	1.549	25.824	82.623
3	0.797	13.283	95.906	1.549	13.283	95.906
4	0.159	2.653	98.559	0.159	2.653	98.559
5	0.086	1.441	100.000	0.086	1.441	100.000
6	−5.053E-16	−8.421E-15	100.000	−5.053E-16	−8.421E-15	100.000

To illustrate the range of the respective original variables represented by each principal component, an analysis was performed using the principal component matrix, as shown in Table 4. PEPC, PK and G6PD show higher indices on the first principal component, indicating that information on these enzymes in the first principal component may act as descriptive indicators of Thr flux; Fructose-1,6-bisphosphate aldolase and MDH show higher indices on the second principal component, indicating that information on these enzymes in the second principal component may act as descriptive indicators of Thr flux. The higher indices shown for HK on the third principal component indicate that the information of this enzyme on the third principal component is able to serve as a descriptive indicator of Thr flux. These three linearly unrelated principal components mainly cover the full range of information and are able to replace the initial six variables.

**TABLE 4:** Matrix of principal components.

Enzyme	Ingredients		
	1	2	3
PEPC	0.946	0.198	−0.007
HK	−0.808	0.051	0.533
FBA	−0.266	0.815	−0.507
MDH	−0.580	0.778	0.218
PK	0.941	0.198	0.099
G6PD	0.754	0.446	0.445

Each score in the score coefficient matrix only represents the correlation coefficient between the principal component and the corresponding variable, which cannot represent the control effect of each enzyme on Thr flux. Under these circumstances, the coefficient matrix corresponding to each indicator in the principal component can be obtained by dividing the data in Table 5 by the corresponding eigenvalue of the principal component and opening the square root. Then the proportion of the eigenvalue corresponding to each principal component to the sum of the total eigenvalues of the extracted principal components was used as the weight to calculate the comprehensive expression coefficient of each principal component, which integrates the control effect of each enzyme on Thr flux. Their values were normalized and recorded as the control coefficient (CCP), as shown in Table 6. G6PD played the most dominant role in controlling Thr flux, while fructose-1,6-bisphosphate aldolase negatively regulated Thr flux. In conclusion, the control coefficients constructed based on quantitative genetic methods reflects the metabolic regulation of Thr flux by the six enzymes.

**TABLE 5:** Matrix of principal component scoring coefficient.

Enzyme	Ingredients		
	1	2	3
PEPC	0.278	0.128	−0.008
HK	−0.237	0.033	0.669
FBA	−0.078	0.526	−0.636
MDH	−0.170	0.502	0.273
PK	0.276	0.128	0.125
G6PD	0.221	0.288	0.558

**TABLE 6:** Comprehensive express coefficient matrix and control coefficients based on principal component analysis.

Enzyme	Principal component expression coefficient matrix			Principal component control expression coefficients	CCP
	A1	A2	A3		
PEPC	0.151	0.103	−0.009	0.116	0.058
HK	−0.128	0.265	0.749	0.1	0.05
FBA	−0.0423	0.422	−0.712	−0.01045	−0.005
MDH	−0.0921	0.403	0.306	0.096	0.048
PK	0.15	0.103	0.140	0.136	0.068
G6DH	0.12	0.231	0.625	0.22	0.11

### Metabolomic analysis of l-threonine synthesis pathway

#### Differential metabolite analysis

With the detection of GC-MS, the metabolites were determined by qualitative and quantitative analyses (Table 7). Organic acids, amino acids, sugars and alcohols represent the main part of metabolites on the aspect of contents. An unreplicated two-way ANOVA was performed to determine whether significant differences exist between strains TWF001 and W3110 in terms of the contents of each metabolite.

**TABLE 7:** Metabolite distribution in experimental and control strains.

Retention time		12 h		24 h		36 h	
(min)	metabolites	W3110	TWF001	W3110	TWF001	W3110	TWF001
7.79	Carbamic acid	0.0423 ± 0.016	0.0843 ± 0.015	0.0668 ± 0.017	0.0468 ± 0.005^a^	0.0667 ± 0.027	0.0542 ± 0.005^b^
8.02	Lactic acid	0.3162 ± 0.027	1.8662 ± 0.26^b^	5.3 ± 0.47	4.1 ± 0.66	4.4571 ± 0.33	2.1569 ± 0.47
8.31	Glycolic acid	0.0061 ± 0.00029	0.0114 ± 0.00304^b^	0.0534 ± 0.007	0.0357 ± 0.006	0.061 ± 0.0037	0.0474 ± 0.01^a^
8.49	Valine	0.0454 ± 0.01764	0.0468 ± 0.024	0.0218 ± 0.015	0.0826 ± 0.031	0.0372 ± 0.1302	0.0329 ± 0.011
8.96	Alanine	0.0477 ± 0.01	0.0527 ± 0.01	0.1164 ± 0.033	0.0203 ± 0.012	0.124 ± 0.03	0.03449 ± 0.005^b^
9.75	Acetic acid	0.0876 ± 0.008	0.1356 ± 0.02	0.0628 ± 0.011	0.1007 ± 0.016	0.0752 ± 0.0025	0.0794 ± 0.017
10.49	Pentanoic acid	0.004 ± 0.0006	0.0688 ± 0.014^a^	0.0189 ± 0.0027	0.081 ± 0.011^b^	0.0144 ± 0.0028	0.0340 ± 0.006
10.65	Leucine	0.0164 ± 0.015	0.0148 ± 0.022	0.012 ± 0.002	0.0095 ± 0.007	0.0127 ± 0.0022	0.0945 ± 0.016^b^
11.42	Ciniic acid	0.0047 ± 0.00056	0.0344 ± 0.00714^a^	0.0073 ± 0.0018	0.0804 ± 0.0272^a^	-	0.0477 ± 0.006
13.75	Thr	0.1033 ± 0.038	5.73 ± 1.16^a^	0.1058 ± 0.046	14.64 ± 1.03^a^	0.0557 ± 0.05	12.1 ± 1.48^b^
13.98	Succinic acid	0.0238 ± 0.00247	0.8841 ± 0.07^b^	0.9643 ± 0.098	1.4598 ± 0.128	0.9193 ± 0.035	0.7538 ± 0.1129^a^
14.49	Glycolic acid	0.0188 ± 0.00654	0.1003 ± 0.00472	0.2145 ± 0.036	0.2412 ± 0.02^a^	0.2056 ± 0.03	0.1332 ± 0.02
14.93	Homoserine	-	0.0913 ± 0.005	-	0.2784 ± 0.05	-	0.2620 ± 0.065
15.40	Malic acid	0.5801 ± 0.05	0.0067 ± 0.001^a^	0.0362 ± 0.0078	0.2784 ± 0.05	0.0528 ± 0.0025^a^	0.0399 ± 0.014
17.52	Citric acid	0.0136 ± 0.0037	0.0248 ± 0.002	0.1563 ± 0.01	0.0383 ± 0.007	0.1640 ± 0.01	0.0322 ± 0.006^a^
18.20	l-Glutamic acid	0.0475 ± 0.0056	0.0129 ± 0.00412	0.8073 ± 0.103	0.0512 ± 0.007^b^	1.5650 ± 0.52	0.0753 ± 0.016^a^
18.44	Sucralose alcohol	0.0067 ± 0.00028	0.0243 ± 0.037^a^	-	0.0168 ± 0.003	-	0.0234 ± 0.002
21.57	Arabinose	0.1378 ± 0.201	0.0556 ± 0.043	0.1337 ± 0.14	0.0717 ± 0.068	0.1197 ± 0.03^a^	0.0437 ± 0.004^b^
24.48	Tartar	0.0281 ± 0.0035	0.0052 ± 0.001	0.0472 ± 0.011	0.0237 ± 0.002^b^	0.0418 ± 0.0042	0.0315 ± 0.011
27.60	Palmitic acid	0.4538 ± 0.15	0.3034 ± 0.09	0.4939 ± 0.127	0.1728 ± 0.08	0.4113 ± 0.188	0.3219 ± 0.114
27.79	Aspartic acid	0.1493 ± 0.02	0.0029 ± 0.00026^a^	0.1425 ± 0.12	0.0152 ± 0.005^b^	0.2632 ± 0.05	0.0201 ± 0.0022^b^
28.82	Inositol	0.0152 ± 0.00072	0.0088 ± 0.0005	0.0347 ± 0.007	0.0173 ± 0.006	0.0237 ± 0.003	0.0177 ± 0.0032
30.56	Stearic acid	0.2889 ± 0.12	0.1933 ± 0.06	0.2829 ± 0.15	0.1345 ± 0.039^b^	0.2882 ± 0.143	0.2281 ± 0.03^a^
32.80	Mannitol	0.0124 ± 0.0008	0.56 ± 0.11^b^	0.045 ± 0.007	0.148 ± 0.013	0.3133 ± 0.017	0.3995 ± 0.026
35.37	Monopalmitin	0.2102 ± 0.039	0.2334 ± 0.063	0.2153 ± 0.11	0.1428 ± 0.07	0.3178 ± 0.1	0.1639 ± 0.1062
37.72	Glyceryl monostearate	0.1450 ± 0.018	0.1636 ± 0.022	0.1193 ± 0.03	0.1281 ± 0.03	0.5383 ± 0.063	0.1409 ± 0.07

Note: Metabolite contents are g L^−1^.

a Refers to *p* < 0.05.

b Refers to p < 0.01.

**FIGURE 4:**
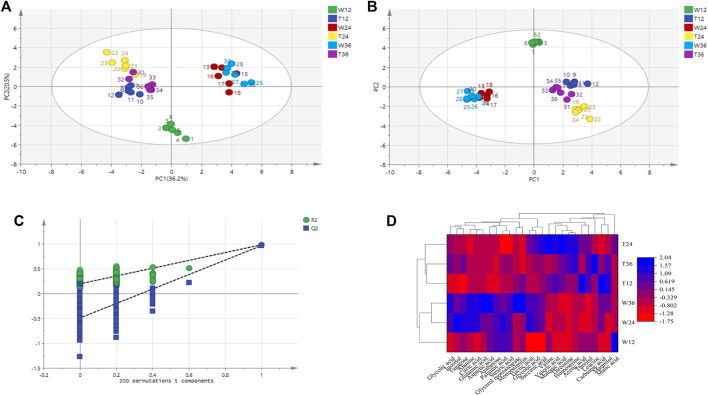
Metabolite difference in strain TWF001 and W3110. **(A)** Score plot of metabolite principal component analysis. **(B)** OPLS-DA score plot of metabolite principal component analysis. **(C)** OPLS-DA displacement test plot. **(D)** Cluster analysis of the two strains. Red represents the zone of low concentration of differential metabolites and blue represents the zone of high concentration of differential metabolites.

Principal component analysis was carried out for the experimental strain (sample T) and the control strain (sample W) and Figure 4A shows that the differences between the two groups of metabolites are significantly obvious. The principal component interpretation rate *R* ^2^(X) = 0.868 > 0.5 indicates a good model fit, and Q^2^ = 0.5651, which is less different from *R* ^2^(X), indicating the stability of the fitted equations. PCA is an unsupervised analysis method that cannot ignore the errors of each group and eliminate random errors, so it is necessary to adopt a supervised approach to identify differences between groups. Orthogonal partial least squares discriminant analysis (OPLS-DA) is a supervised analysis method that combines partial least squares and discriminant analysis. OPLS-DA classifies the sample variable matrix X as both correlated and uncorrelated with Y, and removes the irrelevant variation variables, which enables a comprehensive analysis of between- and within-group variance. From Figure 4B, here *R* ^2^(X) and *R* ^2^(Y) describe the explanatory rate of the model, and Q^2^ = 0.899 represents the predictive power of the model. These three indicators are close to 1, indicating a good reliability of this model (Li et al., 2021). The samples in the Figure 4B are all within the 95% confidence interval and the two class of samples are significantly differentiated with the dispersion in the T sample being greater than the dispersion in the W sample.

An OPLS-DA model with good predictability and fit has been developed, while a 200-response permutation test model was also necessary to be constructed to prevent the model from over-fitting (Figure 4C). Here *R* ^2^(Y) (interpretability of the Y variable) and Q^2^ (model predictability) are important parameters for model evaluation, and their regression lines are crossed with the horizontal coordinates or less than 0, which indicates that the model is relatively accurate. The *R* ^2^(Y) value (0.94) in the OPLS-DA model is approximately equal to 1, indicating that the model is relatively reliable and is able to reflect the real situation of the sample data. The intercept of Q^2^ on the *Y*-axis is negative, and the *R* ^2^ = 0.218 and Q^2^ = -0.483 obtained from the replacement test are smaller than the initial values of *R* ^2^ and Q^2^ in the OPLS-DA model, which suggests that the model is not over-fitted.

The data set was scaled by the heatmap package in Origin 2018 and a cluster analysis was made based on the differential metabolites and their contents to further reflect the metabolic differences between the two strains. Figure 4D shows that the same strain is more correlated even at different culture times, while the metabolic differences between TWF001 and W3110 are high, which is consistent with the results of the principal component analysis.

### Difference analysis of l-threonine metabolic network and potential biomarkers

**FIGURE 5:**
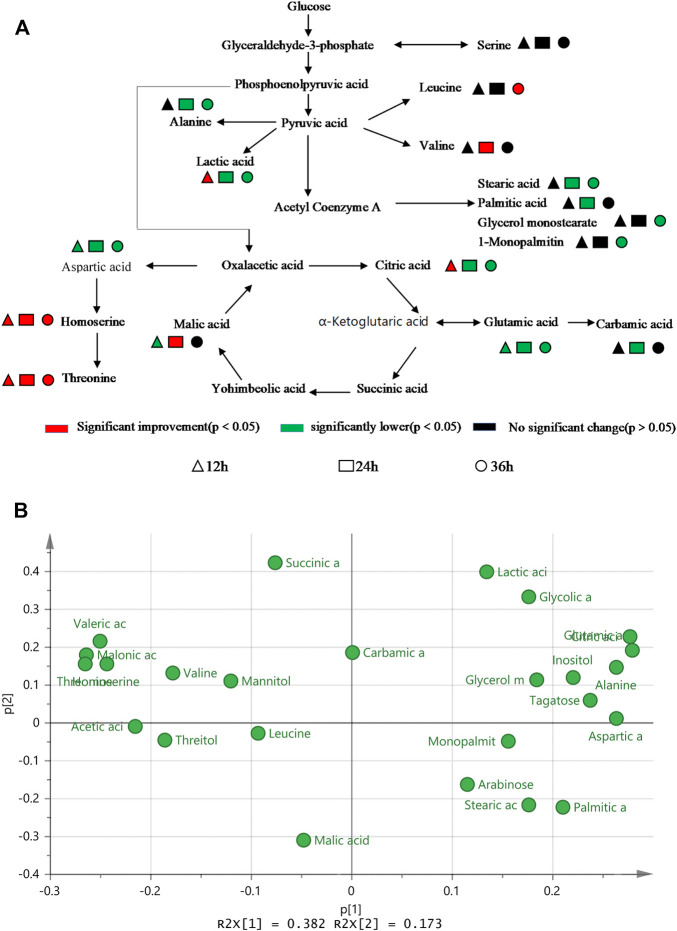
**(A)** Difference in metabolic level between TWF001 and W3110. **(B)** Load plot for metabolite principal component analysis between TWF001 and W3110.

Figure 5A shows that the main metabolites contents that differed between TWF001 and W3110 include amino acids, organic acids, fatty acids, sugars, alcohols and other substances. The contents of l-alanine, l-glutamic acid, l-aspartic acid and fatty acid compounds were significantly lower in TWF001 when compared with the control strain. TWF001 produced higher content of Thr with less carbon metabolic flow to other by-product amino acids. However, l-glutamate plays an important role in the pathway of l-aspartate, the precursor of Thr synthesis, thus up-regulation of the relative genes of glutamate synthesis has several sets of effects on Thr synthesis (Zhao et al., 2020). As a by-product, the increased concentration of l-leucine is detrimental to Thr synthesis, so Lee (Lee et al., 2007)et al. weakened the enzyme activity by site directed mutation of *ilvA* (encoding Thr dehydratase), and thereby reducing l-isoleucine and l-leucine concentrations to prevent Thr degradation. In addition, the higher C flow to by-product amino acids and fatty acid branched pathways in W3110 eventually led to lower Thr level in that strain.

When the intracellular environment is disturbed by the external factors, the metabolic change will occur to adapt to this disturbance and a new metabolic equilibrium is achieved. In PCA analysis, the load diagram represents the magnitude of the contribution made to differentiate between experimental and control groups, with the further away from the origin simply indicating that the metabolite is making a more significant contribution and can be regarded as a biomarker. As shown in Figure 5B, citric acid, l-glutamic acid and inositol are the furthest from the origin, and the levels of these metabolites are proportional to the synthesis of Thr within a certain range. Therefore, these metabolites can be good biomarkers to be added in the medium to regulate the synthesis of Thr.

### A case of metabolic regulation based on metabolic analysis of Thr biosynthesis pathway

**FIGURE 6:**
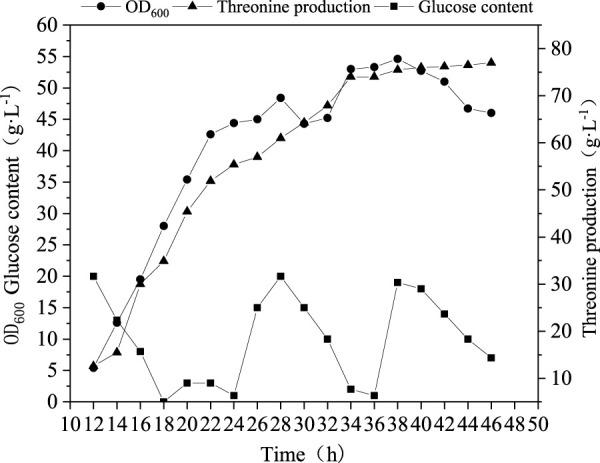
| Profile of Thr production under fed-batch mode with the addition of 8 L-glutamic acid

From above analyses, some key nods, enzymes and biomarkers have been identified as important targets for the production of Thr. Here, we selected addition of l-glutamic acid as an example to verify the reliability of the biosynthesis pathway analyses. l-glutamic acid was added into medium and fed-batch fermentation was conducted to evaluate the performance. As can be seen from Figure 6, the highest OD_600_ value of was not improved, but the Thr production was promoted up to 77 g L^−1^, 10% higher than that without addition of l-glutamic acid. This fact indicates that l-glutamic acid may function by migrating the metabolic flow to Thr rather than enhancing biomass.

Some metabolic regulators, such as glycine, sodium nitrate and sulphate, are activators or inhibitors of key enzymes in the pathway. However, addition of them is often not sufficient to achieve the desired effect. In addition to environmental disturbance, some key enzymes need to be engineered to change the flow of metabolic flux in order to achieve the most reasonable flux distribution for Thr synthesis.

## Data Availability

The original contributions presented in the study are included in the article/Supplementary Material, further inquiries can be directed to the corresponding author.
