# Supplementary material for: Combined metabolic analyses for the biosynthesis pathway of l-threonine in Escherichia coli
Source: Front Bioeng Biotechnol. 2022 Sep 9;10:1010931. doi: 10.3389/fbioe.2022.1010931 (PMC9500239; doi:10.3389/fbioe.2022.1010931)
Supplement: Supplementary file 1 [file DataSheet1.docx]

Supplementary methods

Plackett-Burman (PB) experiment

The PB experimental design is a method of selecting factors from multiple experimental factors that have a significant effect on the experimental index. Based on the previous single factor test results, some important factors were selected. For each factor in the experiment, two levels were taken with the low level being the original level and the high level taking a value of 1.25-2 times the low level. The experimental design is shown in Table S1.

This study was designed using Design Expert 8.0.6 software to create a multiple regression equation. The regression equation reached significance (p < 0.05, ANOVA) and the coefficient of determination R^2^ indicates that the model has a few percent of the variability in the experimental data that could be explained by this regression model.

**Table S1 PB experiment screening experimental factor level change table (g·L^-1^)**

| X | Factors | Low level（-1） | High level（+1） |
| --- | --- | --- | --- |
| X1 | Glucose | 20 | 40 |
| X2 | Ammonium sulphate | 3 | 6 |
| X3 | Magnesium sulphate | 0.4 | 0.8 |
| X4 | Potassium chloride | 0.7 | 0.9 |
| X5 | Betaine hydrochloride | 0.5 | 0.9 |
| X6 | Manganese sulphate | 0.01 | 0.03 |
| X7 | Ferrous sulphate | 0.01 | 0.03 |
| X8 | Phosphate | 0.9 | 1.8 |

**Table S2 Reaction rate equation of metabolic nodes**

| Metabolic node | Reaction rate equation | Metabolic node | Reaction rate equation |
| --- | --- | --- | --- |
| G6P  Xibu5P  Xyl5P  Xib5P  E4P  Sed7P  F6P  GAP  P3G  PEP  Pyr | r1–r2–r8 = 0  r8–r9–r10 = 0  r9–r12–r11 = 0  r10–r11=0 r13–r12–r14=0 r11–r13=0 r2–r3+r13+r12=0 r12+2r3-r4-r13+r11=0 r4-r5=0 r5-r14-r18-r6=0 r6-r7-r16=0 | α-KIV  AcCoA  α-KG  OAA  Asp  Suc  Hom  DAHP  Glu  NADPH | r16–r17=0 r7–r19=0 r19–r22–r20+r23=0 r18+r21–r23–r19=0 r23–r24=0 r20–r21=0 r24–r25–r26=0 r14–r15=0 r22–r17–r23=0 2r8-r22+r7-r23-2r24=0 |

**Table S3 Regression model analysis of variance**

| Factors | Sum of squares | Freedom | Mean Square Error | F | P | Significance |
| --- | --- | --- | --- | --- | --- | --- |
| Models  Glucose  NH_4_SO_4_  MgSO_4_  KCl  Betaine  hydrochloride  FeSO_4_  Phosphate  R^2^  Adj R^2^  Variation coefficient | 0.640  0.025  3.6E-0.03  0.045  1.4E-0.04  0.170  1.9E-0.07  0.048  0.350 | 8  1  1  1  1  1  1  1  1 | 0.640  0.025  3.6E-0.03  0.045  1.4E-0.04  0.170  1.9E-0.07  0.048  0.350  0.969  0.887  23.5% | 11.62  3.67  0.52  6.45  0.021  24.26  2.7E-0.05  6.94  51.10 | 0.034  0.151  0.521  0.085  0.895  0.0160  0.996  0.078  0.006 | *  ** |

* denotes statistical significance (p < 0.05)

** denotes highly statistical significance (p < 0.01)

**Table S4 Variation rate and metabolic flux of metabolites**

| Extracellular detectors | Molecular mass | Accumulation/consumption rate [g·(L·h)^-1^] | | Carbon metabolic flow [mmol·(L·h)^-1^] | |
| --- | --- | --- | --- | --- | --- |
|  |  | 9.8g·L^-1^ Phosphate | 24.8g·L^-1^ Phosphate | 9.8 g·L^-1^ Phosphate | 24.8g·L^-1^ Phosphate |
| Glc  Val  Met  Tyr  Thr | 180  117.15  149.21  181.19  119.12 | 5  0.006  0.027  0.004  2.25 | 2.16  0.16  0.14  0.10  0.60 | 166.70  0.26  0.90  1.80  75.6 | 72  0.20  0.10  2.97  3.92 |
